# Identification of reference genes for qRT-PCR in granulosa cells of healthy women and polycystic ovarian syndrome patients

**DOI:** 10.1038/s41598-017-07346-x

**Published:** 2017-07-31

**Authors:** Yue Lv, Shi Gang Zhao, Gang Lu, Chi Kwan Leung, Zhi Qiang Xiong, Xian Wei Su, Jin Long Ma, Wai Yee Chan, Hong Bin Liu

**Affiliations:** 1 Center for Reproductive Medicine, Shandong University, Jinan, Shandong 250001 China; 2 CUHK-SDU Joint Laboratory on Reproductive Genetics, School of Biomedical Sciences, the Chinese University of Hong Kong, Hong Kong, China; 3 National Research Center for Assisted Reproductive Technology and Reproductive Genetics, Jinan, Shandong 250001 China; 4 SDIVF R&D Centre, Hong Kong Science and Technology Parks, Hong Kong, China

## Abstract

Comparative gene expression analysis by qRT-PCR is commonly used to detect differentially expressed genes in studies of PCOS pathology. Impaired GC function is strongly associated with PCOS pathogenesis, and a growing body of studies has been dedicated to identifying differentially expressed genes in GCs in PCOS patients and healthy women by qRT-PCR. It is necessary to validate the expression stability of the selected reference genes across the tested samples for target gene expression normalization. We examined the variability and stability of expression of the 15 commonly used reference genes in GCs from 44 PCOS patients and 45 healthy women using the GeNorm, BestKeeper, and NormFinder statistical algorithms. We combined the rankings of the three programs to produce a final ranking based on the geometric means of their stability scores. We found that *HPRT1*, *RPLP0*, and *HMBS* out of 15 examined commonly used reference genes are stably expressed in GCs in both controls and PCOS patients and can be used for normalization in gene expression profiling by qRT-PCR. Future gene-expression studies should consider using these reference genes in GCs in PCOS patients for more accurate quantitation of target gene expression and data interpretation.

## Introduction

Polycystic ovarian syndrome (PCOS) is characterized by ovulatory dysfunction, polycystic ovaries, and hyperandrogenism and is a common gynecological endocrinopathy and a leading cause of female reproductive failure^1, 2^. Its prevalence is estimated to be 6% to 18% depending on the diagnostic criteria used^3–5^. PCOS patients are prone to multiple metabolic disorders, including insulin resistance, type 2 diabetes mellitus, obesity, dyslipidemia, chronic low-grade inflammation, cardiovascular diseases, and psychological disturbances^1–3, 6–11^.

While the etiopathogenesis of PCOS remains elusive, it likely involves aberrant hormonal responses mediated by ovarian granulosa cells (GCs) during the progression of follicular development. Previous studies have reported that PCOS is associated with elevated luteinizing hormone (LH) and overexpression and overactivation of the luteinizing hormone/chorionic gonadotropin receptor (LHCGR) in GCs from PCOS patients and that this prevents follicle maturation and ovulation^12–16^. Impaired GC function is associated with disruption of folliculogenesis and with elevated intraovarian androgens and circulating anti-Müllerian hormone levels^17–23^.

Comparative gene expression profiling in normal and disease samples provides invaluable insights into the underlying mechanism of PCOS pathogenesis. Quantitative real-time PCR (qRT-PCR), microarray and RNA-seq have been widely used to measure differential gene expression of GCs from metabolically normal subjects and PCOS patients to identify the genetic regulators and their associated pathways as well as to identify biomarkers in PCOS^24–35^. Most studies have simply selected a reference gene, e.g. *β*-actin (*ACTB*)^36, 37^, 18 S ribosomal RNA (*RNA18S5*)^38^, peptidylprolyl isomerase B (*PPIB*)^39, 40^, or glyceraldehyde-3-phosphatedehydrogenase (*GAPDH*)^41^, as an endogenous internal control for target gene expression normalization without careful examination of their expression variability and stability in the tested samples. Accumulating evidence suggests that these reference genes, previously thought to be stably expressed with minimal variability across samples, are in fact variably expressed under different physiological and pathological conditions^42, 43^. The adoption of an inappropriate housekeeping gene could result in under- or overestimation of gene expression and subsequent misinterpretation of the data^44–46^. To the best of our knowledge, there are no published data that can be used to select reliable reference genes for gene expression normalization in the GCs of reproductively healthy women and PCOS patients.

Given the crucial role of GCs in PCOS pathology, we examined the expression stability and variability of the following 15 commonly used reference genes: *RNA18S5*, *ACTB*, *GAPDH*, ubiquitin C (*UBC*), *β*-2-microglobulin (*B2M*), RNA polymerase II subunit A (*POLR2A*), hypoxanthine phosphoribosyltransferase 1 (*HPRT1*), *ζ*-tyrosine 3-monooxygenase/tryptophan 5-monooxygenase activation protein (*YWHAZ*), hydroxymethylbilane synthase (*HMBS*), *β*-glucuronidase (*GUSB*), importin 8 (*IPO8*), phosphoglycerate kinase 1 (*PGK1*), ribosomal protein lateral stalk subunit P0 (*RPLP0*), transferrin receptor (*TFRC*), and ribosomal protein L13a (*RPL13A*) in GCs from healthy control subjects and PCOS patients. This study thus provides valuable insights into the selection of appropriate reference genes for gene expression profiling in GCs from PCOS patients.

## Results

### Clinical and biochemical parameters of the study subjects

The clinical and biochemical parameters of the 44 PCOS patients and 45 healthy controls are presented in Table 1. There were no significant differences in the serum concentrations of estrogen, progesterone, prolactin, or FSH for the PCOS patients and the age-matched healthy controls. As expected, the BMI of the PCOS group was significantly higher than the control group by 7.8% (PCOS: 24.20 ± 4.73 kg/m^2^, control: 22.45 ± 2.99 kg/m^2^, 95% CI: −1.771 to 9.411, *t* = 1.358 *df* = 87, *p* < 0.05). The PCOS group also exhibited 33.9% higher levels of testosterone compared to the control group (PCOS: 36.12 ± 17.6 ng/dl, control: 23.87 ± 7.77, 95% CI: 6.540 to 17.96, *t* = 4.264 *df* = 87, *p* < 0.0001). Although the FSH levels were indistinguishable between the two groups, both LH and the LH/FSH ratio were strongly increased in the PCOS group by 36.4% and 39.8%, respectively (PCOS LH: 8.10 ± 3.87 IU/L, control LH: 5.1 ± 1.84 IU/L, 95% CI: 1.688 to 4.232, *t* = 4.624 *df* = 87, *p* < 0.0001; PCOS LH/FSH: 1.33 ± 0.74, control LH/FSH: 0.80 ± 0.29, 95% CI: 0.2942 to 0.7658, *t* = 4.467, *df* = 87, *p* < 0.0001).

**Table 1:** Anthropometric and clinical data of PCOS patients and healthy controls.

	Control (n = 45)	PCOS (n = 44)	*p*-value
Age (years)	29.64 ± 2.73	28.59 ± 3.20	0.09
BMI (kg/m^2^)	22.45 ± 2.99	24.20 ± 4.73	0.04*
E2 (pg/ml)	35.27 ± 10.66	39.09 ± 15.49	0.17
P (ng/ml)	0.62 ± 0.23	0.67 ± 0.39	0.48
PRL (ng/ml)	18.66 ± 9.63	17.69 ± 9.51	0.63
T (ng/dl)	23.87 ± 7.77	36.12 ± 17.6	<0.001*
FSH (IU/L)	6.54 ± 1.28	6.26 ± 1.51	0.35
LH (IU/L)	5.14 ± 1.84	8.10 ± 3.87	<0.001*
LH/FSH	0.80 ± 0.29	1.33 ± 0.74	<0.001*

BMI: body mass index; E2: estrogen; P: progesterone; PRL: prolactin; T: testosterone; FSH: follicle stimulating hormone LH: luteinizing hormone. *p < 0.05 indicates statistical significance.

### Expression levels of the reference genes

**Figure 1:**
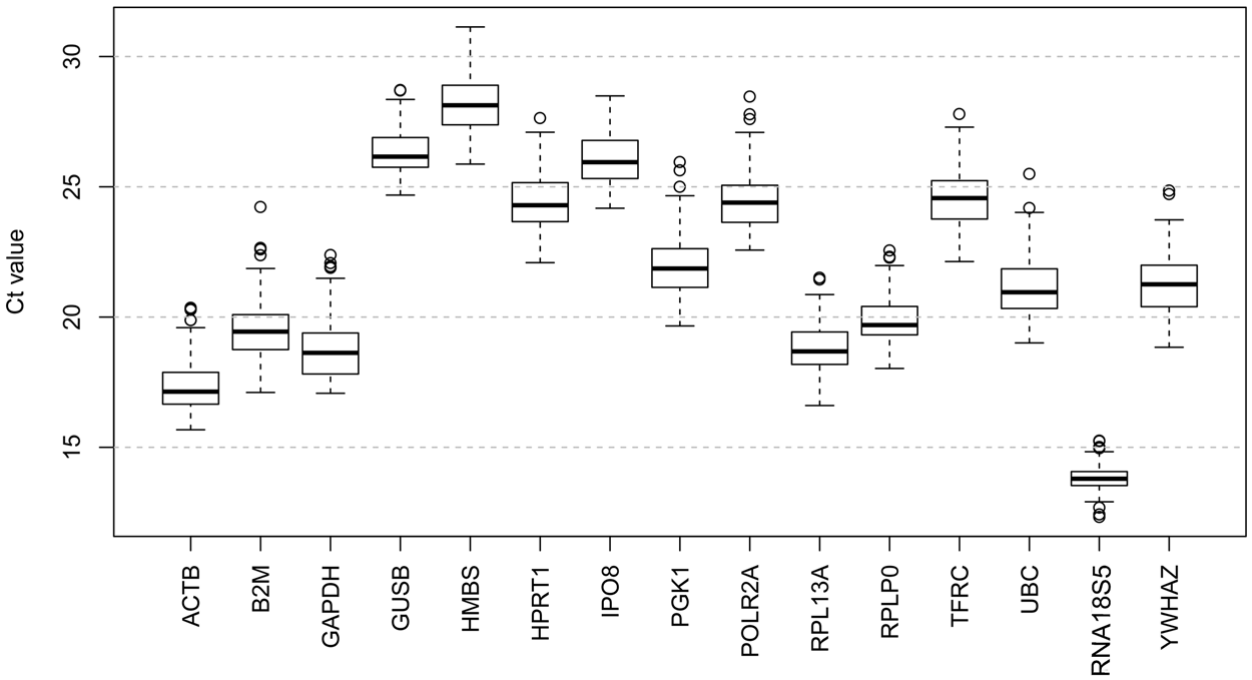
Distribution of threshold cycle (*Ct*) values for the 15 candidate reference genes. Box-and-whisker plots showing the range of *Ct* values for each reference gene. The black center line indicates the median *Ct*. The extended upper and lower hinges indicate 75^th^ and 25^th^ percentiles. The whiskers indicate the largest or smallest *Ct* values falling within 1.5 times the interquartile range from the upper and lower hinges. Small circles indicate the outliers.

The expression levels of the 15 candidate reference genes in all 44 PCOS patients and all 45 controls were evaluated based on their qRT-PCR threshold cycle value (*Ct*). As shown in Fig. 1, their *Ct* values exhibited a dynamic expression range from the lowest of 13.81 ± 0.54 for *RNA18S5* to the highest of 28.25 ± 1.13 for *HMBS* with a median *Ct* value of 21.35 ± 1.12. Among the 89 samples, *RNA18S5* and *ACTB* were the most abundant genes, followed by *GAPDH*, while the least abundant gene was *HMBS*. The assessed reference genes showed significant inter-individual variations across the samples, with *SD* values ranging from 0.54 for *RNA18S5* to 1.26 for *UBC*. Thus it is clear that a vigorous test is required to evaluate the reliability of the 15 candidate reference genes for use as internal controls for comparative gene expression normalization in GCs from PCOS patients.

### Analysis of reference gene expression stability

We used three common algorithms – GeNorm, NormFinder, and BestKeeper – to individually examine the expression stability of the 15 candidate reference genes (Please refer to the Methods).

### GeNorm analysis

**Figure 2:**
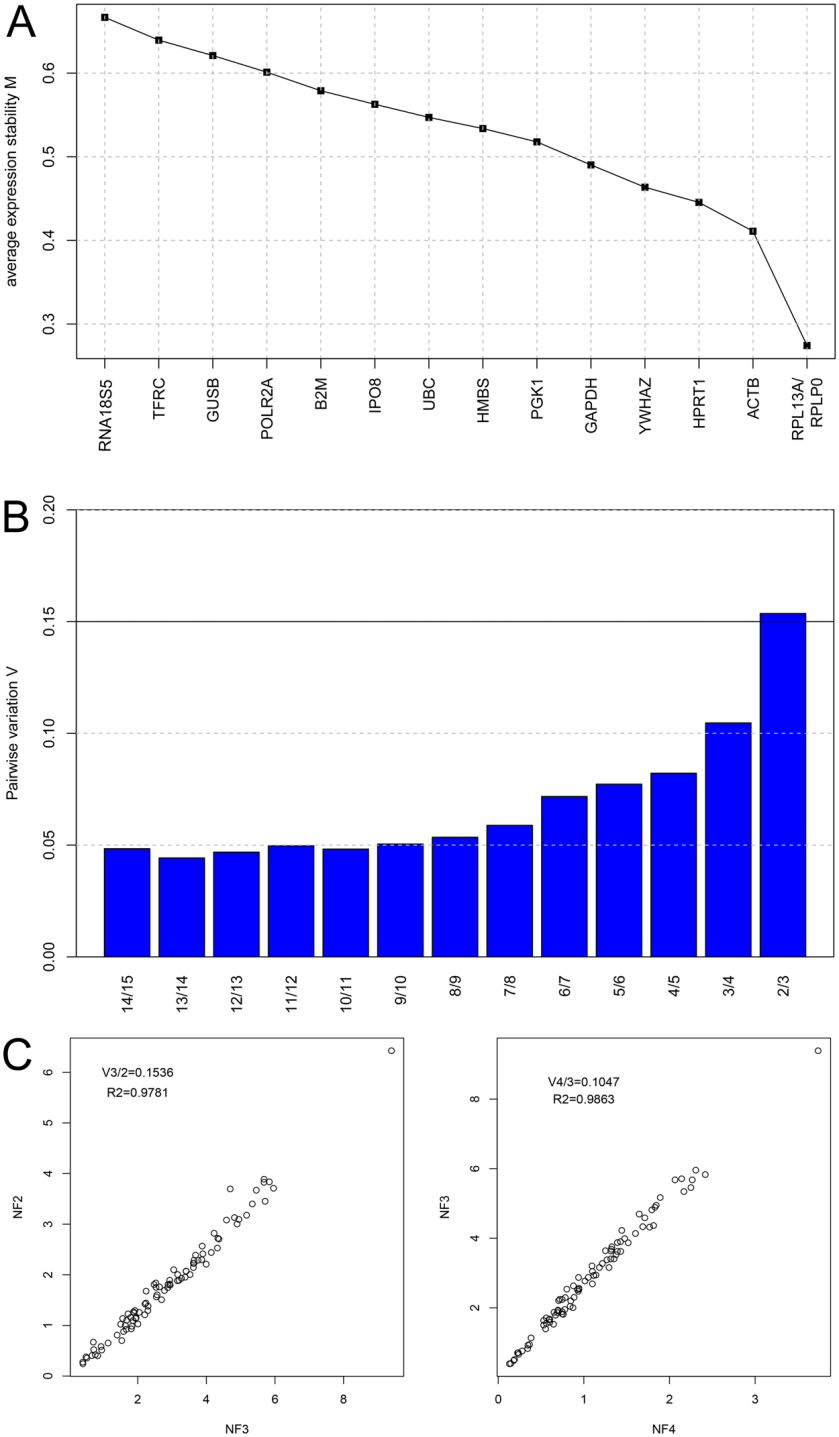
Gene expression stability analysis by GeNorm. (**A**) The mean expression stability value (M) of the 15 candidate reference genes. (**B**) Determination of the optimal number of reference genes for normalization using pairwise variation (*V*_*n*/*n*+*1*_) analysis by GeNorm. (**C**) Selected scatterplots of the normalization factor (NF) before (X-axis) and after (Y-axis) inclusion of an (*n* + *1*)^th^ control gene (*r* ^*2*^ = Spearman coefficient) at which the V-value defines the pair-wise variation between two sequential normalization factors.

GeNorm computed the M-values using a pairwise comparison approach to grade the 15 reference genes based on the similarity of these expression profiles rather than the minimal variation across the 89 samples. Lower M-values indicated greater expression stability. The computed M-values of all of the reference genes were below the defined cut-off value of 1.5, which are considered stably expressed by the algorithm (Fig. 2A). Following the step-wise exclusion of the least stably expressed gene with the highest M-value, the stability values were recalculated. In the pooled group, *RPL13A* and *RLPP0* with the lowest M-values of 0.27 were the most stably expressed gene pair, followed by *ACTB* (0.41), *HPRT1* (0.45), *YWHAZ* (0.46), *GAPDH* (0.49), *PGK1* (0.52), *HMBS* (0.53), *UBC* (0.55), *IPO8* (0.56), *B2M* (0.58), *POLR2A* (0.60), *GUSB* (0.62), *TRFC* (0.64), and *RNA18S5* with the highest M-value of 0.67 making it the least stable gene across all samples.

We also computed the optimal number of reference genes based on the pairwise variation value (V_n/n+1_). GeNorm defines a pairwise variation of 0.15 as the cutoff value, below which (V_n/n+1_ < 0.15) the inclusion of an additional reference gene for normalization is not needed. As shown in Fig. 2B and C, the V2/3 value is just over the cutoff threshold of 0.15 with an expression ratio of 0.1536 (*r* ^*2*^ = 0.978), while the V3/4 value was below the cutoff with an expression ratio of 0.1047 (*r* ^*2*^ = 0.986), indicating that the top three ranked reference genes *RPL13A*, *RLPP0*, and *ACTB* (with the lowest M-values) were suggested for gene expression normalization in GCs from PCOS patients.

### BestKeeper analysis

**Figure 3:**
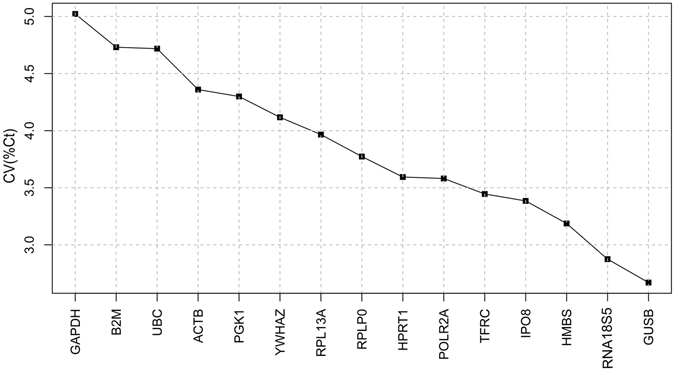
Gene expression stability analysis. Coefficient of variation (%*Ct*) of the 15 candidate reference genes by BestKeeper.

The BestKeeper applet computes the gene expression variation for individual reference genes according to the *SD* and *CV* of their *Ct* values across all of the samples. Figure 3 shows that *GUSB* was ranked the most stably expressed reference gene with a *SD* (±*Ct*) and *CV* (%*Ct*) of 0.70 and 2.67, respectively, followed by *RNA18S5* (*SD*: 0.40, *CV*: 2.87), *HMBS* (*SD*: 0.90, *CV*: 3.19), *IPO8* (*SD*: 0.88, *CV*: 3.38), *TFRC* (*SD*: 0.85, *CV*: 3.45), *POLR2A* (*SD*: 0.88, *CV*: 3.58), *HPRT1* (*SD*: 0.88, *CV*: 3.59), *RPLP0* (*SD*: 0.75, *CV*: 3.77), *RPL13A* (*SD*: 0.75, *CV*: 3.97), *YWHAZ* (*SD*: 0.88, *CV*: 4.12), *PGK1* (*SD*: 0.95, *CV*: 4.30), *ACTB* (*SD*: 0.76, *CV*: 4.36), *UBC* (*SD*: 1.00, *CV*: 4.72), *B2M* (S*D*: 0.93, *CV*: 4.73), and *GAPDH* (*SD*: 0.94, *CV*: 5.02), which was ranked the least stable gene in GCs from PCOS.

### NormFinder analysis

**Figure 4:**
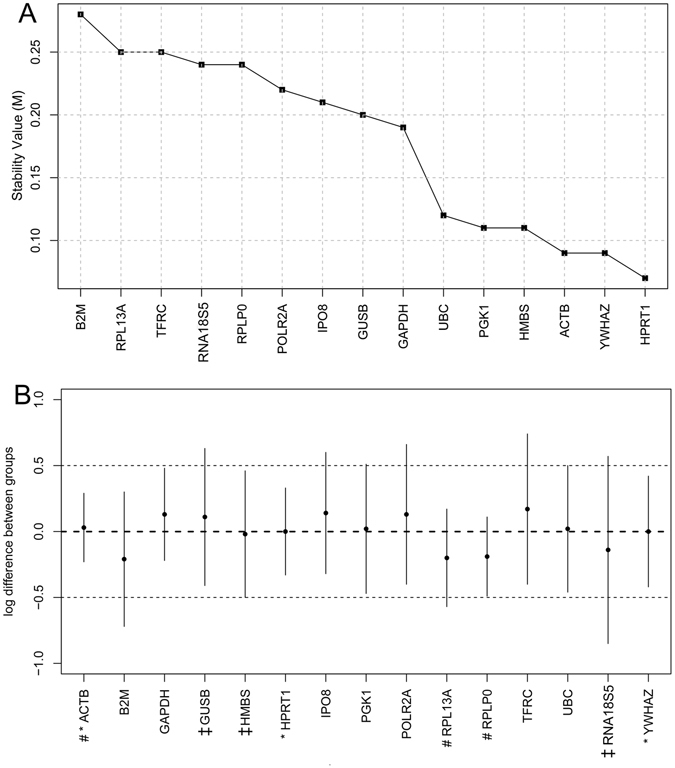
Gene expression stability analysis by NormFinder. (**A**) The mean expression stability value (M) of the 15 candidate reference genes by NormFinder. (**B**) The intergroup variation is plotted (small black circles) as the expression difference between PCOS patients and healthy controls. The vertical bars indicate intragroup variation to provide a confidence interval for the difference. The top three ranked candidate reference genes by GeNorm, BestKeeper, and NormFinder are marked by ^#,^^‡^, and *, respectively, before the gene names on the X-axis. The two thin dashed lines indicate hypothetical target genes with a log difference of 0.5 and −0.5.

We next used NormFinder, a model-based variance estimation algorithm based on the inter-group variation across samples and intra-group expression variation within the same condition to identify the optimal reference gene with minimal variability and coregulation across the samples. As shown in Fig. 4A, in contrast to the GeNorm and BestKeeper results, NormFinder ranked *HPRT1* as the most stable reference gene with an M-value of 0.07, followed by *YWHAZ* (0.09), *ACTB* (0.42), *HMBS* (0.11), *PGK1* (0.11), *UBC* (0.12), *GAPDH* (0.19), *GUSB* (0.2), *IPO8* (0.21), *POLR2A* (0.22), *RPLP0* (0.24), *RNA18S5* (0.24), *TFRC* (0.25), *RPL13A* (0.25), and *B2M*, which, with the highest M-value of 0.28, was ranked as the least stable gene in GCs from PCOS patients.

Figure 4B shows the log difference between groups of the 15 reference genes ranked by the three different algorithms. The model-based NormFinder selected the most stable genes with minimal combined inter- and intragroup expression variation, whereas the pairwise comparison algorithms GeNorm and BestKeeper selected the genes with a low intragroup variation and roughly the same nonvanishing intergroup gene expression variation.

### Final ranking of the 15 reference genes

Given the specific features of the three statistical algorithms, it is not surprising to see marked differences in ranking the 15 candidate reference genes. We used a previously reported method to take into consideration the three sets of results to produce a final ranking of the 15 reference genes^47, 48^. Briefly, the 15 reference genes were re-ranked according to their computed geometric mean from the three algorithms, and a smaller geometric mean indicated higher gene expression stability across all samples. As shown in Table 2, *HPRT1* was the most stably expressed gene with the lowest geometric mean, followed by *RPLP0*, *HMBS*, *YWHAZ*, *GUSB*, *ACTB*, *RPL13A*, *RNA18S5*, *IPO8*, *PKG1*, *GAPDH*, *UBS*, *POLR2A*, *TFRC*, and *B2M*, which was the least stable. Thus *HPRT1*, *RPLP0*, and *HMBS* were the top three ranked reference genes for use as internal controls for qRT-PCR to normalize gene expression in GCs from PCOS patients.

**Table 2:** Final rankings of the 15 candidate reference genes.

Ranking	GeNorm	NormFinder	BestKeeper	Final ranking
1	RPL13A/RPLP0	HPRT1	GUSB	HPRT1
2		YWHAZ	RNA18S5	RPLP0
3	ACTB	ACTB	HMBS	HMBS
4	HPRT1	HMBS	IPO8	YWHAZ
5	YWHAZ	PGK1	TFRC	GUSB
6	GAPDH	UBC	POLR2A	ACTB
7	PGK1	GAPDH	HPRT1	RPL13A
8	HMBS	GUSB	RPLP0	RNA18S5
9	UBC	IPO8	RPL13A	IPO8
10	IPO8	POLR2A	YWHAZ	PGK1
11	B2M	RPLP0	PGK1	GAPDH
12	POLR2A	RNA18S5	ACTB	UBC
13	GUSB	TFRC	UBC	POLR2A
14	TFRC	RPL13A	B2M	TFRC
15	RNA18S5	B2M	GAPDH	B2M

## Discussion

PCOS is a heterogeneous gynecological endocrine disorder characterized by a broad spectrum of anomalies, including chronic anovulation, hyperandrogenism, and hyperinsulinism with insulin resistance, and a growing body of evidence has linked dysfunctional GCs to the pathogenicity of PCOS. To gain insight into the underlying mechanism of impaired GC functions in PCOS development, qRT-PCR has been used extensively to measure differential gene expression in normal and PCOS samples. Most researchers have chosen a reference gene for target gene expression normalization based on the assumption of its inherent expression stability across the tested samples without prior validation. The ideal reference gene requires a constant expression level across all treatments, physiological conditions, tissue/cell types, and experimental designs, but no such reference genes have been identified and they might not even exist^49–56^.

The expressions of the commonly used reference genes in qRT-PCR analyses might vary considerably in specific biological contexts resulting in errors in gene expression estimations. Sadek *et al*. reported that *YWHAZ*, *CYCI*, and *ACTB* were the most stably expressed reference genes out of nine examined housekeeping genes in endometrial tissues collected from PCOS patients, and the commonly used *GAPDH* gene was not recommended^57^. Milutinović *et al*. showed that *HPRT*, in comparison to *ACTB*, *BSM*, and *GAPDH*, was the most stable internal control for normalizations in peripheral blood mononuclear cells in normal and obese women with PCOS^58^. Lindholm *et al*. (2011) examined the expression of a panel of inflammation markers in abdominal superficial subcutaneous tissues from overweight and lean patients with PCOS. They found that *RPLP0* was the best reference gene for target gene expression normalization compared to *LRP10* and *PP1A*. The identification of reliable reference genes for expression normalization in GCs in PCOS patients has not been reported.

In this study, we evaluated 15 candidate reference genes commonly used for routine gene expression normalization in comparative gene expression profiling by qRT-PCR. Given their dynamic expression levels with significant expression variation across the samples (Fig. 1), we used three commonly used statistical programs –GeNorm, BestKeeper, and NormFinder – to analyze their *Ct* values and to rank them according to their M-values. GeNorm indicated that *RPL13A* and *RPLP0* were the two most stably expressed genes with the same computed M-values, followed by *ACTB* (Fig. 2A). The best combination recommended by GeNorm was *RPL13A*, *RPLP0*, and *ACTB* (Fig. 2B,C). In contrast, BestKeeper ranked *GUSB*, *RNA18S5*, and *HMBS* as the top three most stable genes, while *RPLP0*, *RPL13A*, and *ACTB* were ranked at 8^th^, 9^th^, and 12^th^ place, respectively. It is also notable that *RNA18S5* was the least stable gene according to GeNorm (Fig. 3). NormFinder determined *HPRT1* and *YWHAZ* to be the two most stable genes, which were ranked at 4^th^ and 5^th^ place, respectively, by GeNorm and at 7^th^ and 12^th^, respectively, by BestKeeper (Fig. 4). It is worth noting that both GeNorm and NormFinder ranked *ACTB* and *GAPDH*, the two most commonly used reference genes, at the top position; however, they were ranked as the 12^th^ and the least stable genes by BestKeeper (Table 2). The rankings by GeNorm and NormFinder were more consistent with each other than with the BestKeeper algorithm. Finally, we produced the final ranking by considering the results generated by the three programs. We identified *HPRT1*, *RPLP0*, and *HMBS* as the three most stably expressed genes for use as internal controls for comparative gene expression profiling by qRT-PCR (Table 2).

To the best of our knowledge, there is no gold standard for the optimal number of the candidate reference genes required for the expression stability study. In general, six to twelve candidate genes are commonly included for the analysis^59–76^. The supply of cDNA samples and the lab resources dedicated for the characterization should also be considered to make the final decision on the number of candidate genes used for the analysis.

The three identified stably expressed reference controls are specific to our experimental design. It would be of great interest to test the expression stability of these normalizers in the GCs sampled from the PCOS patients from different geographic populations or different species. Nonetheless, our statistical pipelines to identify stably expressed reference genes for comparative gene expression analysis should be broadly applied for general biomedical research.

## Method

### Patient recruitment

Written informed consent was obtained from all participants or their next of kins, and the study was approved by the Institutional Review Board of Reproductive Medicine of Shandong University, and all methods were performed in accordance with the relevant guidelines and regulations. Eighty-nine Chinese Han women aged 29.12 ± 3.01 years were recruited from the Reproductive Hospital Affiliated to Shandong University between January 2015 and June 2016. This study included 44 PCOS patients and 45 women with normal ovulatory function undergoing *in vitro* fertilization (IVF) for tubal and/or male infertility treatment. The study required no modification of our routine IVF protocol.

### Clinical and biochemical measurements

PCOS was diagnosed according to the 2003 Rotterdam criteria^77, 78^, including any two of the following three clinical features: oligo/anovulation, clinical and/or biochemical hyperandrogenism, and polycystic ovaries on ultrasound. Women with other pathophysiological conditions associated with hyperandrogenemia, including adrenal congenital hyperplasia, Cushing’s syndrome, and androgen-secreting tumors, were excluded.

Anthropometric parameters of all subjects, including body height, and weight were measured during the first visit to the outpatient department. The body mass index (BMI) was calculated as weight (kg) divided by the square of the height (m^2^). Venous blood samples were collected between 8:00 a.m. and 10:00 a.m. after a 12 h overnight fast. All blood samples from PCOS patients were obtained during the early follicular phase of their menstrual cycle on days 3–5. The serum levels of follicle-stimulating hormone (FSH), luteinizing hormone (LH), total testosterone, estrogen, progesterone, and prolactin were assayed enzymatically using an automated biochemistry analyzer (Olympus 600, Clinical Chemistry Analyzer, Olympus Diagnostica GmbH, Co. Clare, Ireland).

### Isolation of GCs

Cumulus GCs were isolated from all 44 PCOS patients undergoing IVF and the 45 women in the control group undergoing regular treatment for tubal and/or male factor infertility. Volunteers with a history of other gynecological or medical disorders were excluded. All women were injected with gonadotropin-releasing hormone agonist at the onset of their midluteal phase, and an ultrasound scan and serum estradiol assays were performed to monitor follicle size. When three or more follicles with a mean diameter of R1.8 cm were detected, 8,000–10,000 IU human chorionic gonadotropin (Profasi, Serono) was administered 36 hours prior to the ultrasound-guided immature oocyte retrieval procedure. After anesthetization, the oocyte retrieval was performed through a 17-gauge double-lumen aspiration needle (K-OPS-WOOD-1235, Cook Australia). The cumulus GCs around the oocytes were collected and washed twice with Dulbecco’s modified Eagle medium (DMEM) for subsequent analysis after oocyte removal in follicular aspirates using a Pasteur pipette. Red blood cells were removed with lysis buffer.

### RNA extraction and cDNA synthesis

Total RNA from isolated GCs was extracted using the Trizol Plus RNA Purification kit (Life Technologies) according to the manufacturer’s instructions. The RNA purity was confirmed using a NanoDrop 2000 (Thermo Scientific) and an A260:A280 ratio of 1.9–2.1. Total RNA (1 µg) was reverse transcribed to cDNA using the PrimeScript RT reagent Kit (Takara) and diluted with nuclease-free water to a final volume of 20 µl. The cDNAs were further diluted 1:20 with nuclease-free water for use as the DNA template for qRT-PCR.

### qRT-PCR

We used an epMotion 5075LH (Eppendorf, Germany) automated liquid handling workstation for precise and accurate pipetting of qPCR reagents. The qPCR mix was prepared in a 10 µl final volume containing 5 µl SYBR Green Master Mix, 0.5 µl of primer pair solution (10 µM), 3 µl of nuclease-free water, and 1 µl of the diluted cDNA. The amplification reactions were performed in 384 well plates using PowerUp™ SYBR® Green Master Mix (ThermoFisher) on a QuantStudio™ 7 Flex Real-Time PCR System (Applied Biosystem, USA) according to the manufacturer’s instructions. The thermal cycling conditions were set as follows: uracil-DNA glycosylase (UDG) activation at 50 °C for 2 minutes, DNA polymerase activation at 95 °C for 2 minutes, 40 cycles of denaturation at 95 °C for 15 seconds and annealing/extension at 55 °C for 30 seconds. Specificity of amplification was confirmed by melting curve analysis. The qPCR primers sequences are shown in Table 3.

**Table 3:** qPCR primer set of the 15 reference genes.

Symbol	Gene name	Accession number	Forward primer (5′ → 3′)	Reverse primer (5′ → 3′)
RNA18S5	18S ribosomal RNA	NR_003286.2	GGCGCCCCCTCGATGCTCTTAG	GCTCGGGCCTGCTTTGAACACTCT
ACTB	Actin beta	NM_001101.3	CGACAGGATGCAGAAGGAG	ACATCTGCTGGAAGGTGGA
GAPDH	Glyceraldehyde-3-phosphate dehydrogenase	NM_001256799.2	TGATGACATCAAGAAGGTGGTGAAG	TCCTTGGAGGCCATGTAGGCCAT
UBC	Ubiquitin C	NM_021009.6	ATTTGGGTCGCGGTTCTTG	TGCCTTGACATTCTCGATGGT
B2M	Beta-2-microglobulin	NM_004048.2	TGACTTTGTCACAGCCCAAGATA	CGGCATCTTCAAACCTCCA
POLR2A	RNA polymerase II subunit A	NM_000937.4	GCACCACGTCCAATGACAT	GTGCGGCTGCTTCCATAA
HPRT1	Hypoxanthine phosphoribosyltransferase 1	NM_000194.2	TGACACTGGCAAAACAATGCA	GGTCCTTTTCACCAGCAAGCT
YWHAZ	Tyrosine 3-monooxygenase/tryptophan 5-monooxygenase activation protein, zeta polypeptide	NM_001135699.1	ACTTTTGGTACATTGTGGCTTCAA	CCGCCAGGACAAACCAGTAT
HMBS	Hydroxymethyl-bilane synthase	NM_001135699.1	GGCAATGCGGCTGCAA	GGGTACCCACGCGAATCAC
GUSB	Glucuronidase beta	NM_000181.3	GTCTGCGGCATTTTGTCGG	CACACGATGGCATAGGAATGG
IPO8	Importin 8	NM_001190995.1	TCCGAACTATTATCGACAGGACC	GTTCAAAGAGCCGAGCTACAA
PGK1	Phosphoglycerate kinase 1	NM_000291.3	GAACAAGGTTAAAGCCGAGCC	GTGGCAGATTGACTCCTACCA
RPLP0	Ribosomal protein lateral stalk subunit P0	NM_001002.3	AGCCCAGAACACTGGTCTC	ACTCAGGATTTCAATGGTGCC
TFRC	Transferrin receptor	NM_001128148.2	GGCTACTTGGGCTATTGTAAAGG	CAGTTTCTCCGACAACTTTCTCT
RPL13A	Ribosomal protein L13a	NM_001270491.1	GCCATCGTGGCTAAACAGGTA	GTTGGTGTTCATCCGCTTGC

### Statistical Analyses

The expression stability of the 15 reference genes was analyzed using the three R-based or Microsoft Excel-based algorithms – GeNorm^56^, NormFinder^79^, and BestKeeper^80^. GeNorm selects an ideal reference gene pair whose expression ratios exhibit minimal influence by external factors across different experimental conditions and samples. The first step in the algorithm is to determine the computed stability value (M-value) based on the average pair-wise variation between a gene and all other assessed reference genes as an indication of the stability of the reference gene expression. The gene with the highest M-value is then eliminated, and the selection process will be repeated to select the two most stably expressed genes with the lowest M-values. GeNorm was also used to determine the minimal number of reference genes for gene expression normalization. According to the pairwise variation calculation, for any gene pair with a cutoff (V_n/n+1_) lower than 0.15, an additional (n + 1) reference gene will not significantly improve the normalization and thus is not required for normalization^56^.

NormFinder calculates the variance within and between groups to determine the gene expression stability. Based on the estimate of the intragroup and intergroup stability, a stability value will be assigned for each reference gene. The program ranks all assessed genes based on the M-values, and the gene with the lowest stability is considered the most stably expressed gene.

BestKeeper determines the expression stability according to the coefficient of correlation to the BestKeeper index, which consists of the geometric means of the *Ct* values of the tested gene set. The coefficient of variance (*CV*) and standard derivation (*SD*) are determined, and the reference gene with the lowest *CV* and *SD* is considered the most stably expressed gene. Genes with *SD* > 1 are discarded. This program considers the relationship between the BestKeeper index and the reference genes by calculating the Pearson correlation coefficient, the coefficient of determination (*r* ^*2*^), and the *p*-value.

Numerical values of the clinical characteristics of PCOS cases and controls were expressed as the mean ± *SD*. Independent-samples *t-*tests were performed using SPSS v.19.0 (SPSS Inc., Chicago, IL, USA). A value of *p* < 0.05 was regarded as statistically significant.
